# Promoting Personal Recovery in People with Persisting Psychotic Disorders: Development and Pilot Study of a Novel Digital Intervention

**DOI:** 10.3389/fpsyt.2016.00196

**Published:** 2016-12-23

**Authors:** Neil Thomas, John Farhall, Fiona Foley, Nuwan Dominic Leitan, Kristi-Ann Villagonzalo, Emma Ladd, Cassy Nunan, Sue Farnan, Rosalie Frankish, Tara Smark, Susan L. Rossell, Leon Sterling, Greg Murray, David Jonathon Castle, Michael Kyrios

**Affiliations:** ^1^Centre for Mental Health, Swinburne University of Technology, Hawthorn, VIC, Australia; ^2^Monash Alfred Psychiatry Research Centre, Monash University and The Alfred, Melbourne, VIC, Australia; ^3^Department of Psychology and Counselling, La Trobe University, Melbourne, VIC, Australia; ^4^NorthWestern Mental Health, Royal Melbourne Hospital, Melbourne, VIC, Australia; ^5^Wellways Australia, Melbourne, VIC, Australia; ^6^Department of Psychiatry, St Vincent’s Hospital, Fitzroy, VIC, Australia; ^7^Centre for Design Innovation, Swinburne University of Technology, Hawthorn, VIC, Australia; ^8^Department of Computing and Information Systems, University of Melbourne, Parkville, VIC, Australia; ^9^Department of Psychiatry, University of Melbourne, Parkville, VIC, Australia; ^10^Research School of Psychology, Australian National University, Canberra, ACT, Australia

**Keywords:** schizophrenia, psychosis, personal recovery, mental health services, low intensity interventions, digital health, tablet computers, peer support

## Abstract

**Background:**

For people with persisting psychotic disorders, personal recovery has become an important target of mental health services worldwide. Strongly influenced by mental health service consumer perspectives, personal recovery refers to being able to live a satisfying and contributing life irrespective of ongoing symptoms and disability. Contact with peers with shared lived experience is often cited as facilitative of recovery. We aimed to develop and pilot a novel recovery-based digitally supported intervention for people with a psychotic illness.

**Methods:**

We developed a website to be used on a tablet computer by mental health workers to structure therapeutic discussions about personal recovery. Central to the site was a series of video interviews of people with lived experience of psychosis discussing how they had navigated issues within their own recovery based on the Connectedness–Hope–Identity–Meaning–Empowerment model of recovery. We examined the feasibility and acceptability of an 8-session low intensity intervention using this site in 10 participants with persisting psychotic disorders and conducted a proof-of-concept analysis of outcomes.

**Results:**

All 10 participants completed the full course of sessions, and it was possible to integrate use of the website into nearly all sessions. Participant feedback confirmed that use of the website was a feasible and acceptable way of working. All participants stated that they would recommend the intervention to others. Post-intervention, personal recovery measured by the Questionnaire for the Process of Recovery had improved by an average standardized effect of *d* = 0.46, 95% CI [0.07, 0.84], and 8 of the 10 participants reported that their mental health had improved since taking part in the intervention.

**Conclusion:**

In-session use of digital resources featuring peer accounts of recovery is feasible and acceptable and shows promising outcomes. A randomized controlled trial is the next step in evaluating the efficacy of this low intensity intervention when delivered in conjunction with routine mental health care.

## Introduction

Psychotic disorders represent one of the leading causes of disability and need for ongoing health care in working age adults. In Australia, for example, approximately 4.5 per 1,000 people receive specialist mental health care for a psychotic disorder each year, not including those treated exclusively by private psychiatrists or in primary care (1). In spite of the routine use of antipsychotic medication, and efforts over the past two decades to ensure psychosis is promptly treated at its first emergence, health outcomes remain unsatisfactory for many. For example, the 2010 Australian National Survey of High Impact Psychosis reported that 92% of people seen in specialist mental health services have either recurring or unremitting episodes, 62% experience continuous symptoms, and 90% have deteriorated social functioning (1).

For individuals who experience persisting symptoms and disability, *personal recovery* has become an important target of mental health services internationally (2–7). Often contrasted with the traditional treatment targets of minimizing symptoms (*clinical recovery*) or improving social and occupational functioning (*functional recovery*), the concept of personal recovery has developed from the perspectives of people who use mental health services to prioritize more personal and subjectively meaningful goals of treatment (8–10). A widely used definition is that recovery is “a deeply personal, unique process of changing one’s attitudes, values, feelings, goals, skills and/or roles. It is a way of living a satisfying, hopeful, and contributing life even with limitations caused by the illness” (11). The literature on personal recovery has primarily been based on mental health service consumer narratives about the processes that have been most relevant in their own recovery. Although typically characterized as an individual journey, there has been convergence on identification of processes involved in recovery. An influential synthesis of qualitative studies on recovery has highlighted five themes, summarized by the acronym Connectedness–Hope–Identity–Meaning–Empowerment (CHIME): (C) greater social Connectedness, (H) fostering Hope and optimism, (I) transformation of Identity from one dominated by stigma and a passive patient role, (M) developing new Meaning in life, often deriving meaning from mental health experiences, and (E) Empowerment and responsibility for self-managing mental health (12).

The understanding of processes associated with recovery provides a framework for the development of novel interventions suitable for use in mental health services. This field is at an early stage. Results of the recent REFOCUS trial suggested that delivering training based on the CHIME model to promote recovery-oriented practice in services may have a relatively limited impact on measures of personal recovery (13). On the other hand, positive outcomes for measures of recovery have been found for a number of self-management programs, which include materials on recovery (14–16). Notably, these programs tend to incorporate a strong perspective of learning from shared lived experience, with trialed interventions typically featuring peer co-facilitation and group format delivery, encouraging peer contact and peer-to-peer discussion. Peer-delivered services, peer worker roles, and peer-facilitated interventions have increasingly been a key component of this broader recovery movement (8). In a qualitative metasynthesis of what people find helpful about peer support, peers providing a positive role model, engendering hope, and forming new connections were the key themes (17). Likewise, peer contact is often highlighted as having contributed to recovery in consumer narratives (12), which suggests that hearing directly from others with shared lived experience may be a useful component in recovery-oriented interventions.

In the project reported here [Self-Management and Recovery Technology (SMART)], we developed and piloted a scalable intervention tool suitable for use within mental health services to promote personal recovery. In developing the intervention, we saw potential in creating resources in a digital format that could be incorporated into mental health service consultations using a tablet computer as well as being directly accessible by consumers (18). Initial studies of Internet-based applications with people with persisting psychosis have indicated self-guided use of digital tools to be feasible with this population (19–21). Moreover, the Internet is potentially empowering of people with mental illness in facilitating peer-to-peer connections (22) and is a means of presenting lived experience material in video format that may be useful in portraying positive hopeful views of peers with mental health problems (23). Hence, digital technology offers a number of possibilities for promoting learning from lived experience.

We developed an online intervention tool featuring lived experience accounts of personal recovery as central to a series of modules based on the CHIME framework. This paper presents data on the feasibility and acceptability of using this tool in sessions with a mental health support worker and provides a preliminary examination of outcomes.

## Materials and Methods

### Website Development

The website was developed through parallel processes of end-user consultation; content conceptualization and writing; development of lived experience video materials; and site design. The consultation process included a reference group of seven mental health service consumers with experiences of psychosis who met every 2 weeks during the development phase; a series of focus groups with mental health practitioners from both clinical services and community support services (24), followed by a monthly practitioner reference group; and a further focus group with family carers.

### Content Development

The development of content was an iterative process, combining the conceptual framework of CHIME with input from consultations and emerging content from the filming process. The CHIME framework was used to inform the main content themes, presented as modules as follows:

1. *Recovery*, comprising an introduction to the concept of recovery and aiming to use lived experience material to promote hope and optimism about recovery being possible. As the recommended starting module, this also included guidance on using the site.
2. *Managing Stress*, covering recognition of the relationship between stress and mental health symptoms; identification of common stressors; and coping strategies. This was included as a key element of empowerment in self-managing mental health.
3. *Health*, covering self-management of physical health and medication as a further key element of empowerment, with topics encompassing the link between physical and mental health; making changes in areas such as diet, exercise, sleep, and substance use; and medication.
4. *Me*, covering topics related to identity including the effects of stigma; personal growth through experiencing mental health problems; and focusing on strengths.
5. *Relationships*, covering topics relating to connectedness, including the interaction between interpersonal relationships and mental health; considering the range of contexts in which connections with others can be fostered; nurturing existing relationships; and exploring opportunities for new social connections.
6. *Empowerment*, covering empowerment in interactions with mental health service providers, including material acknowledging the power imbalance experienced in receiving mental health services; how to get the most out of services; and rights and advocacy.
7. *Life*, covering topics related to developing new meaning in life including consideration of the personal values that make life meaningful and identifying related goals.

**Figure 1:**
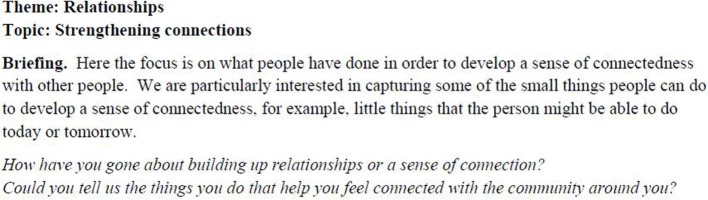
**Example interview question posed to video participants**.

The video material that was central to the site was developed by conducting a series of interviews with persons with lived experience of psychosis using a semi-structured interview derived from the content framework. To develop the interview questions, a working group that encompassed academic, practitioner, and lived experience expertise developed ideas for questions based on topics within the CHIME model for each theme, refined further with reference group input. Questions were developed to draw out the following: (a) how the theme had been relevant to the interviewee’s experience (e.g., the impact of mental illness and associated stigma on how the person saw themselves) and (b) what the person had done to navigate that issue in their own recovery (e.g., ways in which the person had changed how they viewed themselves during recovery). Questions were worded to generate talking from a first person perspective reflecting on their own experiences (e.g., what they had observed from their experiences) rather than from a second person perspective (e.g., advice they would give to a peer about recovery). Each question was preceded by a briefing to orient the participant to the types of material the interviewer was interested in (see Figure 1), with the interviewer actively following up material provided in response, in order to thoroughly explore the topic.

Selected interviewees were first phoned by the interviewer to discuss what participation would involve and to ensure they had thought through the implications of appearing on film. They were then sent information about what their participation would involve and were provided with interview questions a few days prior to filming. To ensure that interviewees felt in control of the experience, it was made clear that the interviewer wanted them to discuss only things that they felt comfortable with. The interviewer revisited whether they were willing for the interview to be used after it had been completed, with the option of having any material deleted if wished. A film crew of two (camera and audio) performed the filming, using a two-camera set-up to facilitate editing, with the interviewer off screen. The interviewer used the semi-structured interview as a guide, but allowed the interview to deviate when useful material was being generated. Questions could be repeated to be refilmed if needed to improve delivery, or align with the first person experiential style of the interviews. At the end of the filming session, each participant was given the option to review a transcript of their interview prior to giving permission for use. Interviewees were paid for their time.

From a pool of 20 potential interviewees who responded to advertising, 11 were selected to form a group with diversity in terms of age, gender, ethnicity, employment status, and sexuality. Interviews were conducted until the material elicited was judged to have reached saturation in its coverage of the content domains; each took approximately 1–2 h.

An extended editing process aimed to generate a series of 2–3 min videos featuring a selection of four to six interviewees discussing key issues for each topic. Given that recovery is characterized as highly individual (12), we aimed to capture different experiences and points of view within each video. Interviews were transcribed, and each line coded by a member of the research team into the various video topics. The combined filmed material for each video topic was reviewed by the content group, with excerpts selected that represented the most useful or impactful material obtained while reflecting a range of experiences and perspectives. A total of 26 videos were produced using this method. Additionally, 11 videos were made introducing each of the peers.

In addition to these lived experience videos, five videos of mental health professionals’ experiences and two videos of family members’ experiences (produced in a similar way) were included as part of material on working with services and relationships. Additionally, 12 videos were produced featuring either a consumer leader or academic expert contributing additional material that elaborated on what was raised in the other videos or by addressing points that had not been captured by the interviews. An introduction to each module was also filmed, featuring a consumer leader as guide. In total 64 videos were included in the final package.

Text content and reflective exercises were added to summarize key points and complement the lived experience content with material from relevant therapeutic approaches (e.g., on implementing coping strategies, on changing health behavior, on identifying personal values).

### Website Design

The design of the site was informed by published guidelines for website development for severe mental illness designed to minimize the impact of difficulties in thinking and memory (25), combined with input from the consultation process, and consideration of how the site could be designed in a way that reinforced recovery. The site was optimized for tablet computer and mobile phone use. Navigation was simplified by organizing content in a minimal number of levels (topic, subtopic), making use of touchscreen scrolling to reduce the required number of page loads and having a single constant menu button. Links between pages were clearly labeled, pages were designed to have minimal distractions, and content was developed to be simple, clear, and logically organized. Key design principles derived from the consultations were (a) simplicity of layout and navigation, (b) flexibility in use (e.g., material can be completed in any order), (c) interactivity, (d) catering to different learning styles and preferences by presenting content in multiple ways, (e) access to any information entered being controlled by the consumer, and (f) promoting a positive emotional experience while engaging with the website (24). Consideration of how recovery processes could be facilitated by the design and features of the site included the following: (a) promoting connectedness by allowing users to comment on material and contribute to a user forum on the site and allowing users to share content with workers, family members, and others; (b) promoting the person taking ownership of their identity by allowing personalization of user profiles and customization of content; and (c) promoting empowerment and responsibility in self-management by the ability to track parameters such as sleep and mood and to set and view goals developed from the material.

**Figure 2:**
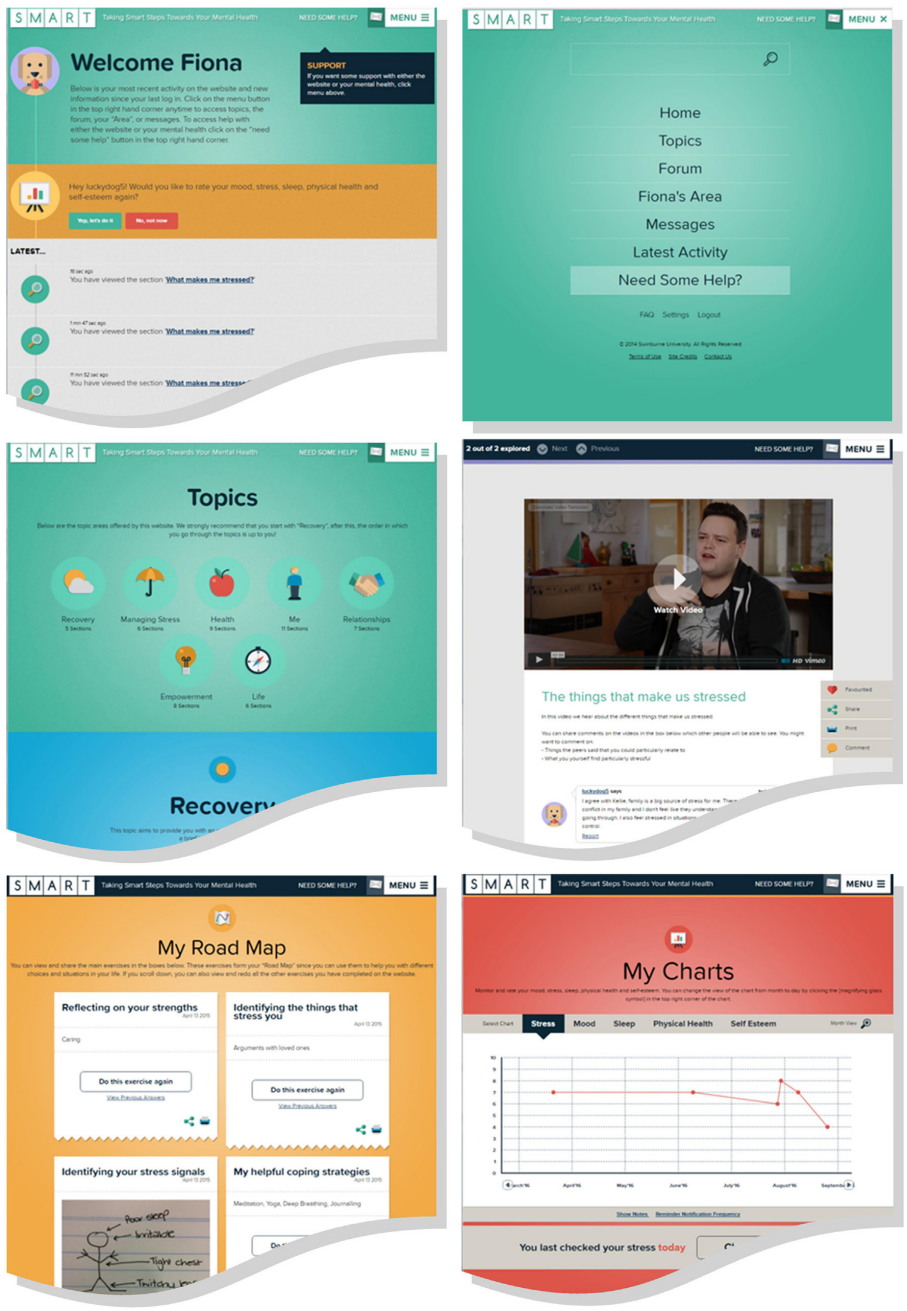
**Example screen shots of SMART online resources**.

The site is accessible only by creation of an account, which enables the user to enter information in reflective exercises, charts, and task lists and to select a username and avatar for posting public comments and using the forum. Forums and comment feeds are monitored by the research team to assess risk of harm to participants, and participants are also able to report any offensive comments for moderator review. Example screenshots are shown in Figure 2.

### Pilot Study

#### Design

To examine feasibility and proof-of-concept of using this tool within service delivery, a pilot study was conducted in the form of a single-arm trial of a mental health worker-facilitated intervention using the SMART website on a tablet computer with participants. All participants received the intervention in addition to treatment as usual during a 3-month delivery window. Assessments were completed at baseline and at 3 months. The project was conducted in accordance with the Declaration of Helsinki, and was approved by the Human Research Ethics Committees at The Alfred (study no. 139-14), St Vincent’s Hospital Melbourne (study no. 041.14) and Melbourne Health (study no. 2014.087). All participants gave full informed consent prior to commencement.

#### Participants

Participants were recruited through a combination of mail-outs of consumers, clinician referral at community mental health services, and presentation to consumers at residential services in metropolitan Melbourne. Inclusion criteria were: (a) aged 18–65 years; (b) diagnosis of a psychotic disorder (schizophrenia-related disorder or bipolar disorder or major depressive disorder with the presence of a severe episode with psychotic features within the past 2 years), confirmed using the Structured Clinical Interview for DSM-IV-TR Axis I Disorders [SCID; (26)]; (c) sufficient fluency in English to make use of the resources; (d) overall intellectual functioning within normal limits, having an estimated IQ over 70 based on the Wechsler Test of Adult Reading [WTAR; (27)]; (e) access to Internet at home or *via* smartphone. Exclusion criteria were (f) organic psychosis; and (g) change in antipsychotic medication, in-patient admission, or the commencement or completion of formal psychological therapy within the preceding 2 months.

#### Procedures

A research assistant met with potential participants to obtain informed consent and complete baseline assessments, including the SCID, WTAR, and first administration of outcome measures. Eligible consenting participants were then provided with a time for their appointment with one of the two facilitators, and went on to complete the eight session intervention. The post-intervention assessment was scheduled for 3 months following the baseline assessment, to allow time for missed sessions. The post-intervention assessment also included a treatment evaluation questionnaire. Following this assessment, participants were also contacted by telephone by the project manager to obtain feedback on the site to refine content, during which they were also asked questions about their experience of the intervention more broadly.

#### Intervention

The intervention consisted of eight 50-min face-to-face sessions with an experienced mental health support worker (facilitator), using a tablet computer from which the SMART website was accessed. Participants were assigned to one of two trained facilitators, seconded from the community mental health support sector, and attended sessions at weekly to fortnightly intervals within a window of 3 months, in addition to the participant’s routine treatment. An account for the participant to use the website was set up during the first intervention session, and they were shown how to access it to facilitate use outside sessions.

Sessions involved the collaborative selection of content from the seven themes on the site, followed by shared viewing and discussion of website material. Discussions included reflecting on the website content as applicable to the participant’s own recovery, considering changes participants may wish to enact based on these reflections, and setting goals for the upcoming week. Facilitators encouraged participants to use the website between sessions, complete reflective exercises, and/or make public posts about the content, if willing.

#### Feasibility and Acceptability

Feasibility and acceptability were indexed by the following:

1. The proportion of sessions in which the website was used, assessed by the site’s record of log-on records during times appointments were held.
2. Participant qualitative feedback from the post-intervention interview on the process of using the site in-session with a worker.
3. The number of participants choosing to access the website between appointments, assessed by the site’s record of log-ons between appointments.
4. Rate of and reasons for dropout from the intervention.
5. Satisfaction with the site, assessed by response to the question “would you recommend the site to other people?” during the feedback interview, coded as affirmative or negative.
6. Acceptability in terms of positive versus negative emotional impact of using the site, assessed by the item “Overall, did the website make you feel better, or worse, or no different?” in the post-intervention questionnaire.

#### Outcome Measures

The following measures were completed pre- and post-intervention to provide a preliminary assessment of the outcome.

##### Personal Recovery

Personal recovery was the primary outcome. The Questionnaire for the Process of Recovery [QPR; (28)], a 22-item self-report measure developed in conjunction with mental health service users to assess personal recovery. The QPR has good psychometric properties (29) and was used because of its strong alignment with the CHIME framework (30). However, because it was a relatively new measure for which sensitivity to intervention effects was unknown, we also included total score on the more established 41-item Recovery Assessment Scale (31) as a second measure of personal recovery.

##### Recovery dimensions

Hope was measured using total score on the Schizophrenia Hope Scale (32), a 9-item questionnaire assessing optimism and hope for the future, rated on 3-point items (*disagree, agree, strongly agree*). Social connectedness was measured using total score on the Friendship Scale (33), comprising six 5-point (*not at all* to *almost always*) items.

##### Psychotic Symptoms

The interviewer-rated Positive and Negative Syndrome Scale [PANSS; (34)] was used to assess the severity of psychotic symptoms and their impact on behavior and functioning. The two research assistants were trained in the standardized administration of the PANSS. In addition, the Subjective Experiences of Psychosis Scale (35) was used to assess the subjective impact of psychotic symptoms in participants reporting ongoing positive symptoms at baseline. This is a 29-item questionnaire on which participants rate the positive and negative impact of their symptoms on aspects of their feelings and behavior, such as “hope for the future” and “ability to socialize.” Subjective impact is rated on a 5-point scale from *not at all* to *very much* in the past week. The negative impact subscale score was used in analyses.

##### Emotional Symptoms

The 21-item Depression Anxiety Stress Scale [DASS-21; (36)] total score and its associated subscales were used to assess emotional symptoms. Participants reported on their experience of symptoms related to depression, anxiety, and stress in the past week, on a 4-point scale from *did not apply to me at all* to *applied to me very much or most of the time*.

##### Quality of Life

Total score on the Assessment of Quality of Life-8 Dimension [AQoL-8D; (37)] was used to assess health-related quality of life. This 35-item questionnaire encompasses eight dimensions of physical and psychosocial health, with lower scores indicating fewer issues related to subjective quality of life in the past week.

#### Process Measures

It was hypothesised that the lived experience content of the intervention would influence outcome by increasing self-efficacy for positive recovery, and by reducing the extent to which mental illness is viewed in negative stigmatized terms. The proposed mechanism was assessed by the following measures.

##### Self-Stigma

Self-stigma was used as an index of negative views of illness, measured using the 29-item Internalized Stigma of Mental Illness Scale [ISMI; (38)] which includes subscales of alienation, stereotype endorsement, discrimination experience, social withdrawal, and stigma resistance. Items are rated on a 4-point scale from *strongly disagree* to *strongly agree*.

##### Self-Efficacy

Self-efficacy was measured using total score on the Generalized Self-Efficacy Scale (39), a 10-item questionnaire rated on a 4-point scale from *not at all true* to *exactly true*.

Additionally, qualitative feedback on experiences of using the lived experience videos was collated from the post-intervention interview. Use of antipsychotic medication was also recorded at baseline and 3 months as a potential confound.

#### Treatment Evaluation

Subjective perceptions of the helpfulness or otherwise of the intervention were assessed by the item “Do you feel that using the website made the impact of your mental health problems better, or worse, or no different?” based on an item used by our group in previous trials with this population (40, 41). This was rated on a 5-point scale from *much worse* to *much better*. Additionally, 11 5-point Likert items were developed to assess whether participants endorsed changes having occurred in relation to material covered on the site (e.g., “I feel more hopeful about my recovery”). Questions were also included in the qualitative phone interview to gather feedback on use of the site during sessions with a worker, and on specific site elements including the videos.

#### Statistical Analyses

In this pilot study, the emphasis was on examining acceptability and feasibility, as well as allowing a preliminary estimate of treatment effects, rather than using inferential statistics to hypothesis–test specific outcomes. Correspondingly, effect sizes and confidence intervals were calculated for the mean pre- to post-intervention change score. Standardized effect sizes were calculated by dividing the mean change by the average SD of pre and post-intervention scores or, if variances were unequal, the baseline SD (42). A series of paired *t*-tests was also conducted to indicate where two-tailed significances fell within *p* < 0.05. Complete data were available for all participants.

## Results

Twelve potential participants were recruited for the study. Two of these were excluded at baseline: one due to a recent medication change and the other due to participation in another research project. Ten participants completed the baseline assessment (nine males; mean age 42.6 years, SD 12.47, range 23–62 years; six single, four divorced). Nine had a diagnosis of schizophrenia, and one of schizoaffective disorder. None worked full time; two were in paid part-time work and one in volunteer work. All were receiving antipsychotic medication. At baseline, seven participants reported using the Internet at least daily, one once per week, and two “rarely or never.” Half of the participants reported that they “rarely or never” used the Internet to access information about mental health.

### Feasibility and Acceptability

#### In-Session Use

The website was used in 76 of 80 sessions that were attended. Of the remainder, two were initial sessions spent assisting participants setting up email to use the site, and one was a final session consolidating the program material without use of the site. There was only one session in which the support worker was not able to use the site with the participant. Session notes indicated that the participant’s engagement had been threatened in a previous appointment, when the facilitator misunderstood something that the participant was discussing, and subsequently directed them to an unrelated topic on the site. The session was spent having a broader discussion of recovery without use of the site to re-engage the participant, and the participant and the worker went on to use the site further in their remaining sessions.

Qualitative feedback on the use of the site in-session is collated in Table 1. Responses suggested that the process of integrating the website as a tool in sessions functioned well. Some participants expressed that they would have been less engaged or unable to use the site independently without the facilitator sessions, and a number of participants commented that the site facilitated discussion with the worker.

**Table 1:** **Participants’ feedback about using the site together with a worker**.

P1. I think the technology was, it was a guide, it kept our discussion going in a direction that we wanted it to go in. And it would raise the topic or it would raise the, the next discussion, so it was guiding what we were going through. But we did do a lot of talking with [the facilitator] and I felt the two worked together really, really well … perfect. I did say to [her] though that I felt that the facilitator was needed. I felt that while I was at home I didn’t have, there was no accountability. I didn’t have anyone looking over my shoulder telling me “You must do that” and “you must go to the website”, “you must do a module or whatever”, there was no accountability. But with a facilitator where I’m going to see [her], you know, within a few days, and we were going to discuss this, then there was accountability, I had to get some things done.

P2. Yeah it was very easy and [the facilitator] explained everything well to me, yeah. And ah, even, I think even just without her I think I could have gone through it myself it would have been quite easy … yeah, yeah. There was some bits in there, um some bits that were a bit difficult to understand, yeah. Not much, but there were two or three parts that she helped me with … And not only that, ah, having someone there as a support to go through every single one of them, I think that very helpful … I guess the SMART, the website goes into more detail into aspects of my life.

P3. We were able to acknowledge and cover things in more depth than I would have by myself.
P4. It’s fine, so the iPad was useful, but I’m not the type of person to sort of sit down and do that sort of stuff. I’m more of an interactive person with whoever I’m talking to. … I enjoyed talking to her more than using the iPad.

P5. I thought it worked well. Yeah. … Like with the iPad, I thought that, what do you want to discuss today? There’s always, it was more, this program, it was more about what you had to say and what you thought of situations instead of, instead of feeling intimidated when you go in to other ways to see a worker or feeling like oh what are going to say and then feeling like intimidated. But in this case I didn’t feel intimidated, I knew [the facilitator] well, and I thought that she did a great job just explaining everything to me, patience, and all of it.

P6. I thought it was really easy. Really easy. Smooth and, ah yeah, just a pleasant experience … . if it was just sort paper and pencils, it sort of got a bit dull after a while. … but the iPad and the website made it quite colourful and a bit more interesting.

P7. Well I didn’t use it, I know it’s going to sound funny, but we didn’t use it much, just for me to get to where I’d written it at home and then read it out to her, “oh this is what I’ve written and this is what I’ve written, and this is what I’ve done,” and then discuss it because I can’t type properly on an iPad and I like to type really fast and you know, and be able to check my spelling and everything so I just did it at home., and I was happy. … I’d just log on and do some stuff at home and then in the sessions they were really just to go over what I’d done at home and what had come up and so she was sort of acting therapist, poor [facilitator]. … No with the iPad, because I got to share all, everything I’d written, and we’d talk about what I wrote. Talk about subjects and say, “What subject should I do next?” and “What’s that involve?” And, “Maybe this one would work,” and then I’d say, “Can you do a print out of the PDF, and yadayadayada.” A lot more involved than just seeing a therapist, you know what I mean.

P8. [Without the website] we wouldn’t have had nearly as much to talk about. And then I would have been more stuck for words I think. I wouldn’t have been able to talk about all the issues that we had discussed about the website so it would have been a bit more difficult I think.

P9. It was just really good. A good experience with [the facilitator] and the iPad.

P10. I would’ve really hated it if I did it by myself, because I probably wouldn’t have got there anyway, I mean anywhere, but that’s why I thought, I didn’t mind it so much, because um people like [the facilitator] were just such a good guide. But something like, with computers, I couldn’t do it myself, even though it seemed pretty simple, once [the facilitator] was showing me what to do, I just said, I really would hate to do it by myself …

#### Between-Session Use

Six of the 10 participants independently logged on to the site outside of sessions, with a median of 4.5 log-ons among those who did this (range 1–14). Among these six participants was one of the participants who “rarely or never” used the Internet at baseline, the remainder being daily users. Six participants posted public comments on the site, with a median of two posts among those who posted (range 1–20 posts).

#### Drop Out and Satisfaction

All 10 participants attended the full course of eight intervention sessions, and all 10 participants said during the post-intervention interview that they would recommend the site to others.

#### Emotional Impact of Site Use

No participants indicated a negative emotional impact of use of the site, with all 10 reporting a positive effect of using the site on how they felt (responses *better* or *much better*).

### Outcomes

Estimated effect sizes on the outcome measures are presented in Table 2. On the primary outcome of personal recovery, an estimated medium effect size was observed on the QPR, which in spite of the small sample size was statistically significant. A similar magnitude effect was observed on the RAS as a second measure of recovery (also statistically significant). Among other outcomes, medium effects were estimated for the subjective negative impact of psychosis symptoms, emotional symptoms on the DASS, and hope, but there was a negligible effect on social connectedness. A small effect size was estimated on the PANSS and the AQoL-8D.

**Table 2:** **Estimated effects on outcome measures**.

Measure	Mean (SD)		Change score		Effect size		*p*
	Pre	Post	Mean	95% CI	*d*	95% CI	
**Personal recovery**							
QPR	57.50 (11.65)	62.90 (11.89)	5.40	[0.87, 9.93]	0.46	[0.07, 0.84]	0.024
RAS	154.10 (13.59)	163.20 (18.80)	9.10	[1.44, 16.76]	0.56	[0.09, 1.04]	0.025
**Recovery dimensions**							
SHS	17.60 (3.92)	19.80 (6.41)	2.20	[−0.515, 4.92]	0.56	[−0.13, 1.25]	0.10
Friendship Scale	16.00 (2.87)	16.22 (4.66)	0.22	[−3.25, 3.70]	0.08	[−1.13, 1.29]	0.89
**Psychotic symptoms**							
PANSS total	65.70 (18.58)	61.40 (19.51)	−4.30	[−12.51, 3.91]	−0.23	[−0.66, 0.21]	0.27
PANSS positive	17.90 (8.05)	15.70 (5.87)	−2.20	[−4.65, 0.25]	−0.32	[−0.69, 0.03]	0.07
PANSS negative	14.50 (3.60)	14.80 (7.33)	0.30	[−4.42, 5.02]	0.06	[−0.81, 0.91]	0.89
PANSS general	33.30 (10.12)	32.90 (9.43)	−0.40	[−3.91, 3.10]	0.04	[−0.40, 0.32]	0.80
SEPS negative impact	80.43 (30.84)	61.14 (18.87)	−19.29	[−43.82, 5.25]	−0.78	[−1.76, 0.21]	0.10
**Emotional symptoms**							
DASS total	25.20 (16.71)	16.90 (11.21)	−8.30	[−16.91, 0.31]	−0.60	[−1.21, 0.02]	0.06
DASS depression	8.60 (5.72)	6.50 (5.40)	−2.10	[−6.46, 2.26]	−0.38	[−1.16, 0.41]	0.31
DASS anxiety	7.60 (5.19)	5.20 (2.57)	−2.40	[−5.32, 0.52]	−0.46	[−1.03, 0.10]	0.10
DASS stress	9.00 (6.60)	5.20 (4.59)	−3.80	[−6.79, −0.81]	−0.68	[−1.21, −0.14]	0.018
**Quality of life**							
AQoL-8D total	88.20 (17.25)	83.90 (16.58)	−4.30	[−12.01, 3.41]	−0.25	[−0.71, 0.20]	0.24
**Process measures**							
GSES total	27.80 (3.46)	28.40 (5.44)	0.60	[−2.36, 3.56]	0.13	[−0.53, 0.80]	0.66
ISMI total	65.70 (13.48)	61.20 (13.60)	−4.50	[−9.28, 0.28]	−0.33	[−0.69, 0.02]	0.06
ISMI alienation	15.70 (3.89)	13.20 (3.33)	−2.50	[−4.09, −0.91]	−0.69	[−1.13, −0.25]	0.006
ISMI stereotype endorsement	11.80 (3.52)	11.60 (3.57)	−0.20	[−1.14, 0.74]	−0.06	[−0.32, 0.21]	0.64
ISMI discrimination	12.00 (3.23)	11.50 (3.47)	−0.50	[−1.53, 0.53]	−0.15	[−0.46, 0.16]	0.30
ISMI social	15.00 (3.74)	14.40 (3.75)	−0.60	[−2.15, 0.95]	−0.16	[−0.58, 0.25]	0.41
ISMI stigma	11.20 (2.10)	10.50 (1.72)	−0.70	[−2.13, 0.73]	−0.37	[−1.12, 0.38]	0.30

*N = 10, except for SEPS negative impact (N = 7)*.

*QPR, Questionnaire for the Process of Recovery; RAS, Recovery Assessment Scale; SHS, Schizophrenia Hope Scale; PANSS, Positive and Negative Syndrome Scale; SEPS, Subjective Experience of Psychosis Scale; DASS-21, Depression Anxiety Stress Scale 21; AQoL-8D, Assessment of Quality of Life-8 dimension; GSES, Generalized Self-Efficacy Scale; ISMI, Internalized Stigma of Mental Illness*.

Among process measures, a small to medium effect was estimated on self-stigma, but negligible effects were evident on self-efficacy. Examination of subscales of the ISMI self-stigma measure suggested that the strongest effects were in reducing perceived alienation with an estimated moderate to large effect size (statistically significant), while the estimated effect on other domains, such as negative stereotype endorsement, were negligible. Two participants had increases and three had decreases in antipsychotic medication dose during the trial, but changes on the personal recovery measures were not correlated with changes in chlorpromazine-equivalent dose.

In response to the question “Do you feel that using the website made the impact of your mental health problems better, or worse, or no different?” eight participants reported that their mental health was better or much better, with the remaining two reporting it was no different. Agreement was also high on all items of the treatment evaluation questionnaire (Table 3). Specific feedback on the lived experience videos is collated in Table 4.

**Table 3:** **Participant responses on the treatment evaluation questionnaire**.

Since starting SMART …	Number agreeing or strongly agreeing (*N* = 10)
I understand more about my mental health	8
I feel more connected with people	7
I feel more hopeful about my recovery	9
I have progressed in my personal recovery	9
I have a stronger sense of my identity	8
I have a better idea about what my values are	8
I feel more confident about making plans	8
I feel more confident about my rights	8
I feel more confident about working with services	9
I feel more confident about managing my stress	8
I feel empowered to improve my physical and mental health	10

**Table 4:** **Participants’ feedback about lived experience videos**.

P1. I enjoyed, I guess, what’s the name of the word, the reinforcement, or, seeing somebody else going through the same situation with the same feelings, somebody I could relate to, I found, it was something I hadn’t been through before and, and that made me feel good, that felt great and a lot more at ease from watching the video. The video also … what I did try and do once or twice was to answer questions without watching the video and then, going back watching the video, realised that the video was actually opening up the scope, it was actually scoping out the area ahead of what the questions were going to be like.

P2. The fact that others are sharing their own experience. … Yeah, and I look at the video and even though I was hospitalised before, when I come out – it’s been a while since I came out – I forget that ah, I’m not alone. Yeah.

P3. Really enlightening. Made me feel like I am not alone.

P4. Well … relate to people … what they’re saying: this is what happened to me and how I got over it, and what I did. Yeah.

P5. I like the fact that everyone’s so different, it’s so, like they all have different, and they’re all unique, and they all had good things to say. Like what I mean by good is, you know, relevant to people with like, yeah. I didn’t feel so alone. So that was a good thing. … I just felt like I could relate to someone. I wasn’t so alone.

P6. There were obviously the different individuals who explained their scenario and talked about each topic in the video, and then said what that topics means to them, and how certain questions around the topic are answered, and it was all good, it was all insightful. … Yes, the videos were really good, they were organised, they were structured, quite informative, honest, and um yeah, so like lots of multi-perspectives on topics – yeah, that was good.

P7. Because I could just sit there and watch a whole half hour of them talking and get so inspired, and so moved, you know. … it makes you realise you’re not alone, and that you’re not some frumpy sort of, the bad image of mental illness: not washed, not clothed well, smells bad, can’t coherently keep a sentence together, looks off into the distance, is aggressive or threatening or sullen. You know what I mean?

P8. I could relate to a lot of peoples’ stories, and they had a similar experience to mine, so I thought that was good. And then I answered a few questions and sent a few comments to [the facilitator] and that sort of thing. So yeah I just, I gained more insight into my condition I think. I’ve always had a lot of insight, but just hearing other peoples’ experiences; when you think you’ve got your own mind made up about your illness and you won’t listen to anybody about your illness, and you need to think “oh okay,” you think you’re right, but there are a lot of other people who have varying symptoms, and it was just good to hear other peoples’ opinions and impressions of their own diagnosis, and that sort of thing; what they do to tackle their problems. So it was good.

P9. I can relate to some of the things they were talking about in my own life, and it just makes me more aware and more determined to overcome the obstacles that I’ve been facing.

P10. They had people talking about how to handle stress, and I put my feet in their shoes and sort of could understand, you know, um where those people were coming from, their experiences.

## Discussion

This study examined the feasibility of an intervention targeting personal recovery in psychosis, a domain for which intervention development is a priority. It involved the novel combination of lived experience-based content on recovery, presentation *via* a digital medium, and delivery integrated with face-to-face mental health sessions. Overall, it appeared feasible to deliver an intervention in this way, and there were promising findings on the primary outcome of personal recovery.

Our attempts to develop the main content of the site by using a process of editing together various lived experience interviews showed this to be a feasible approach. We used 11 lived experience speakers to provide diversity in age, gender, ethnicity, and sexuality within the group, which was sufficient to produce material for all topics. Not all content within the topics was covered in this way, but complementary scripted videos from experts and text material was used to complete an effective website in which the dominant content was explicitly authored by peers. While many online interventions include “client perspective” videos to illustrate other material [e.g., Ref. (43, 44)], this is the first online intervention, we are aware of, that has an explicit focus on lived experience material as the main vehicle for change.

Use of the site in-session appeared to be feasible and acceptable to participants. The website was used regularly in-session, no participants dropped out, and participants gave generally positive feedback about how the use of the site integrated with face-to-face work. It appeared from participant feedback that many would have found it harder to use or maintain engagement with the site had it not been integrated with sessions with the facilitator. The feedback is consistent with the broader digital mental health literature, where therapist-assisted interventions tend to be engaged with for longer than self-guided interventions (45). However, lower levels of use of digital technology (46) and higher rates of disability among persons with severe mental illness suggest some people may be more reliant upon support to utilize online materials. A blended approach offers a means of capitalizing on the scalability of digital interventions to deliver quality structured interventions, while bearing in mind the barriers to independent Internet use experienced by this group. Indeed, although most participants used the site between sessions as well, which was encouraged, not all of the participants in this pilot did so, suggesting that a significant proportion of people in this population would be reliant on in-session use. Indeed, it should also be noted that most of our sample were daily Internet users at baseline, so their existing levels of computer use were higher than average for this population (46). Given our vision for the website as a vehicle to facilitate discussion between the consumer and worker about recovery, the modest between-session use was not problematic. A number of participants’ responses confirmed that use of the site did help them discuss issues which otherwise might not have been raised or would have been difficult to raise.

Video-based tools may have benefits beyond the present aim of facilitating discussions about recovery: future research could investigate whether patient–worker interactions around embarrassing or sensitive topics (e.g., discussing ambivalence about medication) are supported by tools of this kind. The technology may have broader applications in practice, such as in promoting supported decision making [e.g., Ref. (47)], or as a tool for formal psychological therapies.

As a preliminary proof-of-concept study with a small sample, analysis of outcome was not designed to hypothesis–test efficacy, but to establish whether estimated effects were in a range suggesting full scale trialing to be worthwhile. While we cannot be certain that other ongoing interventions had no impact on outcomes, results were promising, with a moderate effect size being estimated on both measures of recovery that were used. Feedback from participants was also consistent with the intervention having a beneficial impact upon recovery, and participant feedback additionally identified no negative effects. The estimated effect size on recovery is similar to effects observed for other psychosocial interventions for persisting psychosis (48). Together, these findings suggest value in conducting a larger-scale controlled trial of this intervention (49).

## Author Contributions

All authors participated in the development and pilot of the digital intervention. NT, FF, NL, JF, EL, CN, SF, LS, and GM designed the website and developed its content. NT, JF, FF, RF, and TS developed the therapist intervention protocol. NT, K-AV, and FF conducted analyses and prepared the manuscript. All the authors read and approved the final manuscript.

## Conflict of Interest Statement

The authors declare that the research was conducted in the absence of any commercial or financial relationships that could be construed as a potential conflict of interest.
